# Real-World Medical Device Reports of SpaceOAR Hydrogel Spacer: Analysis of the Food and Drug Administration Manufacturer and User Facility Device Experience (MAUDE) Database

**DOI:** 10.1016/j.adro.2025.101824

**Published:** 2025-06-07

**Authors:** Lara Hathout, Young Eun Shin, Sirikan Rojanasarot, Emmanuel Ezekekwu, Danielle Vannan, Michael R. Folkert

**Affiliations:** a Rutgers Cancer Institute, Rutgers Robert Wood Johnson Medical School, New Brunswick, New Jersey; b Pharmacy Practice & Administrative Sciences, James L. Winkle College of Pharmacy, University of Cincinnati, Cincinnati, Ohio; c Health Economics & Market Access, Boston Scientific, Marlborough, Massachusetts; d Medical Affairs, Boston Scientific, Marlborough, Massachusetts; e Department of Radiation Oncology, University of Washington School of Medicine, Seattle, Washington

## Abstract

**Purpose:**

Increasingly, polyethylene glycol (PEG) hydrogel spacers are being used to help reduce rectal toxicity in men undergoing radiation therapy for prostate cancer. Device-related issues and patient event rates from the Manufacturer and User Facility Device Experience (MAUDE) were assessed to contextualize spacer reports in the United States.

**Methods and Materials:**

PEG hydrogel spacer medical device reports (MDRs) from January 1, 2015, through December 31, 2023, were collected from the MAUDE database, which reports suspected device-associated malfunctions, injuries, and deaths submitted to the United States Food and Drug Administration. United States sales data during the same period were obtained from the manufacturer. Reported rates of device-related and patient events were calculated by dividing MDRs by total units sold, recognizing that not all units sold are necessarily placed.

**Results:**

A total of 1005 unique MDRs reported from the United States for PEG hydrogel spacers were identified in the MAUDE database, and 251,836 spacer units were sold during the study period. Five deaths were reported, representing 0.002% of total units sold. The annual MDR rate increased from 0.00% in 2015 to 0.57% in 2023 (overall rate 0.40%). The most frequently reported device-related events were placement/positioning problems (0.295%) and adverse events without an identified device or use problem (0.084%). The most frequently reported patient events were “no clinical signs, symptoms, or conditions” or asymptomatic (0.175%), pain/discomfort (0.110%), infection (0.052%), fistula formation (0.037%), and hemorrhage/blood loss/bleeding (0.033%).

**Conclusions:**

With respect to estimated device usage in the United States, overall device-related events and patient event rates for PEG hydrogel spacers consistently remained below 1%, with the majority having minimal or no reported clinical consequence.

## Introduction

Definitive radiation therapy for prostate cancer is associated with excellent disease-free survival rates^1^; however, treatment morbidity including rectal toxicity can have a significant long-term impact on quality of life and may limit the dose of tumor-controlling radiation therapy administered.^2^, ^3^, ^4^, ^5^, ^6^, ^7^, ^8^, ^9^ The placement of perirectal spacers can facilitate the delivery of high doses of radiation to the target tissues by creating additional space between the prostate and rectum. By displacing nearby normal healthy tissues [ie, organs-at-risk (OARs)] and increasing radiation dose falloff, morbidity following radiation therapy can be significantly reduced.

First cleared by the United States (US) Food and Drug Administration (FDA) in 2015,^10^ biodegradable polyethylene glycol (PEG) hydrogel spacers (SpaceOAR Hydrogel) are intended to temporarily displace the anterior rectal wall away from the prostate during radiation therapy in patients with prostate cancer.^11^, ^12^, ^13^, ^14^, ^15^, ^16^ SpaceOAR PEG hydrogel spacers maintain space for about 3 months and then undergo hydrolysis, resulting in absorption in most cases by approximately 6 months.^14^^,^^17^ Iodinated PEG hydrogel spacers (SpaceOAR Vue Hydrogel), cleared by the FDA in 2019, consist of resorbable iodinated hydrogel radiopaque material, improving visibility of the spacer on computed tomography imaging.^18^

Perirectal spacers are increasingly used in prostate cancer radiation therapy treatment to reduce the radiation dose delivered to the anterior rectal wall.^3^^,^^19^ They continue to be adopted as a standard-of-care procedure for patients undergoing radiation therapy, supported by the existing clinical evidence and ongoing study of their real-world utilization. The FDA’s Manufacturer and User Facility Device Experience (MAUDE) database plays a role in continuous postmarketing surveillance of medical devices, including SpaceOAR.^20^ Over the last decade, several studies have been conducted on SpaceOAR medical device reports (MDRs) using this database, but they feature methodological limitations that may impact their findings. For example, a study by Fernandez et al.^21^ provided an extensive review of MDRs up to October 2022, but did not calculate overall rates of adverse events. Another study by Millot et al.,^22^ despite covering a broader timeframe, omitted individual adverse event rate calculations and included duplicate reports, which may provide an inaccurate portrayal of event frequencies. To develop a more comprehensive understanding of adverse events potentially associated with SpaceOAR, the objectives of this study were to report on the rate of SpaceOAR MDRs in the US after FDA clearance; and provide context of the complications for evaluating the clinical significance of MDRs for SpaceOAR.

## Methods

### Study period and population

MDRs reported to the MAUDE database between January 1, 2015 and December 31, 2023 were used for this study. MAUDE is a passive, postmarketing surveillance system created and managed by the FDA that collects information on adverse events, device malfunctions, and product problems related to medical devices.^20^ The MAUDE database comprises mandatory MedWatch reports from manufacturers, distributors, user facilities, and importers, and voluntary reports from health care professionals, consumers, and patients. Yearly US sales data for SpaceOAR and SpaceOAR Vue were obtained from the manufacturer.^23^ The events data used were retrieved from a publicly accessible FDA database and do not contain patient information; therefore, neither institutional review board approval nor participant consent was obtained.

The MAUDE database was queried using “SpaceOAR” in the brand name to include both SpaceOAR Classic and Vue or “hydrogel spacer” or “perirectal spacer” in the generic name. Hereafter, the term “SpaceOAR” will be used to refer to both SpaceOAR and SpaceOAR Vue. Records reported by past and current manufacturers of the product were included. While the query encompassed MDRs reported globally, the subsequent study analysis focused on reports that identified the source country as “US.” To ensure conservative estimations, MDRs with missing or unknown originating countries were assumed to be all US MDRs.

### Data analysis

Data cleaning was performed using R Statistical Software (v.4.3.2) and data analyses were performed using Microsoft Excel. Reports that were identical in terms of MDR report key, report number, dates the report was received and reported, event type, reporter country, brand and generic names, and reported device and patient event codes were identified as duplicates and removed from the analysis. All reports included were analyzed for frequency characteristics, including report source, reporter country, event type, and device-related issues and patient events. MDR reported rates of device-related issues and patient events, determined by dividing the total US MDRs identified in MAUDE for SpaceOAR by the total spacer units sold in the US between 2015 and 2023, were also reported. Total US units sold of SpaceOAR and SpaceOAR Vue obtained from the manufacturer were used as a proxy for estimating the number of patients who received the spacers. These sales figures do not directly equate to the actual number of patients treated with the spacers, as the true case volume is not available. Spacers units sold may not ultimately be placed because of procedure cancellations, patient clinical issues, unit expiration, and other reasons. While sales data are not linked to patient diagnoses, both SpaceOAR and SpaceOAR Vue are FDA-cleared specifically for use in patients undergoing radiation therapy for prostate cancer.^24^^,^^25^ As a sensitivity analysis to aid in providing conservative estimates of the overall MDR rate, the global MDRs that include both US and non-US reports were divided by the total spacer units sold in the US during the same study period to provide an overall MDR rate. Additionally, the event descriptions for reported deaths were reviewed and detailed to provide context for their reports.

## Results

### MDR characteristics

A total of 1005 unique MDRs reported from the US were identified among 1209 MDRs reported globally for SpaceOAR in the MAUDE database over the 9-year study period. A total of 251,836 SpaceOAR and SpaceOAR Vue units were sold in the US in the same period, yielding a crude MDR rate of 0.40% over the study period. The characteristics of the MDRs are presented in Table 1. Findings were consistent for both SpaceOAR and SpaceOAR Vue. The vast majority of the reports were submitted by the manufacturer (99.2%). Nearly two-thirds of the reports were reported as device malfunctions (65.27%), whereas approximately one-third were related to injuries (34.33%).

**Table 1:** Characteristics of medical device reports (MDRs) for SpaceOAR using US only

Characteristics	Total MDRs (n = 1005)	SpaceOAR	SpaceOAR Vue
Reporter source (*n*, %)			
Manufacturer	997 (99.20)	466	531
Voluntary	8 (0.80)	6	2
Event type (*n*, %)			
Malfunction	656 (65.27)	269	387
Injury	345 (34.33)	201	144
Death	5 (0.50)	3	2

Five deaths were reported during the study period, representing 0.002% of units sold over the study period. With respect to the reported deaths that could possibly have been related to the spacer and/or the spacer placement procedure, one occurred in 2018, following a high-dose-rate brachytherapy procedure and SpaceOAR placement; the patient developed a prostatic abscess and later died from alcoholic cardiomyopathy, with medical evaluation stating that the infection was not believed to be related to SpaceOAR. In 2019, a patient underwent a procedure to place low-dose-rate brachytherapy seeds and SpaceOAR; he experienced dizziness and nausea followed by cardiac arrest immediately after the procedure. In 2020, a patient experienced cardiopulmonary arrest after the procedure, with imaging performed during his workup that showed the spacer was appropriately localized. In 2022, a patient experienced a vasovagal event followed by cardiac arrest after an aborted spacer placement procedure (event occurred on withdrawal of the ultrasound probe). In 2023, a patient again experienced cardiac arrest after the procedure, preceded by an event consistent with a vasovagal reaction (nausea, lightheadedness).

### MDR frequency and rates

The number of MDRs and the MDR reported rate for SpaceOAR increased each year since initial clearance in 2015 (Table 2). The annual MDR reported rate increased from 0.00% in 2015 to 0.57% in 2023. The overall MDR reported rate for the entire study period was 0.40%. The reported rate using global MDR reports (Table E1) was 0.48%. Similar patterns of MDR frequency were observed for SpaceOAR and SpaceOAR Vue. Note that an MDR may include more than one device-related issue or patient event.

**Table 2:** Yearly medical device reports (MDRs), units sold, and reported rates for SpaceOAR

	Total			SpaceOAR			SpaceOAR Vue		
Year	MDR	Units sold	Rate (%)	MDR	Units sold	Rate (%)	MDR	Units sold	Rate (%)
2015	0	1802	0.00	0	1802	0.00	–	–	–
2016	2	5544	0.04	2	5544	0.04	–	–	–
2017	3	9890	0.03	3	9890	0.03	–	–	–
2018	11	22,225	0.05	11	22,225	0.05	–	–	–
2019	65	31,675	0.21	63	31,675	0.20	2	–	–
2020	95	37,155	0.26	90	34,463	0.26	5	2692	0.19
2021	230	48,110	0.48	142	30,568	0.46	88	17,542	0.50
2022	340	50,218	0.68	94	16,840	0.56	246	33,378	0.74
2023	259	45,217	0.57	67	8903	0.75	192	36,314	0.53
Total	1005	251,836	0.40	472	161,910	0.29	533	89,926	0.59

The 10 most frequently reported device-related events for SpaceOAR are presented in Table 3. The reporting of a placement/positioning problem was most common (0.295%), followed by an adverse event without identified device or use problem (0.084%), a material integrity problem (0.007%), a defective device (0.006%), an obstruction of flow problem (0.005%), and use of device problem (0.005%). Device-related events for SpaceOAR and SpaceOAR Vue were similar.

**Table 3:** Ten most frequently reported device-related issues for SpaceOAR between 2015 and 2023

	Total		SpaceOAR		SpaceOAR Vue	
	MDR	Rate (%)	MDR	Rate (%)	MDR	Rate (%)
Positioning problem	743	0.2950	315	0.1251	428	0.1700
Adverse event without identified device or use problem	212	0.0842	130	0.0516	82	0.0326
Material integrity problem	18	0.0071	6	0.0024	12	0.0048
Defective device	14	0.0056	4	0.0016	10	0.0040
Obstruction of flow	12	0.0048	6	0.0024	6	0.0024
Use of device problem	12	0.0048	7	0.0028	5	0.0020
Contamination/decontamination problem	11	0.0044	4	0.0016	7	0.0028
Appropriate term/code not available	10	0.0040	5	0.0020	5	0.0020
Migration	8	0.0032	2	0.0008	6	0.0024
Inadequacy of device shape and/or size	7	0.0028	2	0.0008	5	0.0020

*Abbreviation*: MDR = medical device report.

Note: the order of device-related issues is based on the total number of MDRs.

The 10 most frequently reported patient events are presented in Table 4. The most common event, “no clinical signs, symptoms, or conditions,” signifying that the patient did not experience any clinically apparent issue (no patient complaints such as pain, and no clinically observed consequences) reported in the MDR, occurred in 0.175% of cases. Other common patient events included pain/discomfort (0.110%), infection (0.052%), fistula formation (0.037%), and hemorrhage/blood loss/bleeding (0.033%) (Table 4). The most frequently reported patient events for positioning problems were no clinical signs, symptoms, or conditions (0.166%), pain/discomfort (0.063%), fluid discharge (0.019%), hemorrhage/blood loss/bleeding (0.018%), and urinary dysfunction (0.017%) (Table 5). The 10 most frequently reported patient events for positioning problems were consistent for SpaceOAR and SpaceOAR Vue.

**Table 4:** Ten most frequently reported patient events for SpaceOAR between 2015 and 2023

	Total		SpaceOAR		SpaceOAR Vue	
	MDR	Rate (%)	MDR	Rate (%)	MDR	Rate (%)
No clinical signs, symptoms or conditions	440	0.1747	129	0.0512	311	0.1235
Pain/discomfort	278	0.1104	145	0.0576	133	0.0528
Infection	132	0.0524	75	0.0298	57	0.0226
Fistula	92	0.0365	59	0.0234	33	0.0131
Hemorrhage/blood loss/bleeding	83	0.0330	47	0.0187	36	0.0143
Urinary dysfunction	77	0.0306	47	0.0187	30	0.0119
Ulcer/ulceration	71	0.0282	39	0.0155	32	0.0127
Fluid discharge	71	0.0282	37	0.0147	34	0.0135
Inflammation	62	0.0246	29	0.0115	33	0.0131
No consequences or impact on patient	45	0.0179	40	0.0159	5	0.0020

*Abbreviation:* MDR = medical device report.

**Table 5:** Most frequently reported patient events for positioning problems reported for SpaceOAR.

	Total		SpaceOAR		SpaceOAR Vue	
	MDR	Rate (%)	MDR	Rate (%)	MDR	Rate (%)
No clinical signs, symptoms, or conditions	419	0.1664	297	0.1179	122	0.0484
Pain/discomfort	158	0.0627	81	0.0322	77	0.0306
Fluid discharge	47	0.0187	25	0.0099	22	0.0087
Hemorrhage/blood loss/bleeding	46	0.0183	24	0.0095	22	0.0087
Urinary dysfunction	43	0.0171	21	0.0083	22	0.0087
Ulcer/ulceration	40	0.0159	20	0.0079	20	0.0079
No consequences or impact on patient	37	0.0147	3	0.0012	34	0.0135
Infection	37	0.0147	20	0.0079	17	0.0068
Fistula	36	0.0143	14	0.0056	22	0.0087
Inflammation	28	0.0111	15	0.0060	13	0.0052

*Abbreviation:* MDR = medical device report.

## Discussion

The goal of rectal spacing, regardless of the medical device or technique, is to reduce the radiation dose received by the anterior rectal wall at the level of the prostate, in turn helping to reduce the risk of acute and/or chronic rectal adverse effects. From the dose escalation study reported by Kuban et al. that established 78 Gy in 39 fractions as the standard conventional radiation dose for prostate cancer, grade ≥2 gastrointestinal (GI) toxicity was 26% at 10 years, with grade 3 toxicity seen in 7% of patients.^5^ From this study, dose-volume parameters were derived suggesting that limiting the volume of rectum receiving > 70 Gy by at least 25% would help reduce grade ≥2 GI complications. Although the use of intensity modulated radiation therapy has increased the ability of radiation treatment planning to meet tighter rectal dosimetric constraints, significant rates of rectal toxicity persisted. A meta-analysis of randomized trials primarily using intensity modulated radiation therapy in which rectal spacing was not used reported 5.4% to 22% late grade ≥2 GI toxicity for conventionally fractionated radiation therapy, 8.9% to 25.6% for hypofractionated radiation therapy, and 10% for ultra-hypofractionated radiation therapy (stereotactic body radiation therapy dosing), respectively.^26^ A pivotal prospective multicenter randomized controlled trial by Mariados et al. evaluated the outcomes following spacer implantation. A total of 222 patients received conventional radiation therapy to 79.2 Gy in 44 fractions, with or without hydrogel spacer placement.^14^ Overall, acute grade ≥2 rectal toxicity did not differ but late grade ≥2 rectal toxicity was 1.4% in the control arm compared to 0% in the rectal spacer arm. At a median follow-up of 3 years, toxicity outcomes on both arms were excellent; late grade ≥2 rectal toxicity remained lower in the rectal spacer arm, with no grade ≥2 rectal events observed in the rectal spacer arm compared to 5.7% in the control arm.^12^ This study resulted in the FDA clearance of hydrogel spacers for standard use in patients receiving external beam radiation therapy for prostate cancer.

Analyses from the current study show that, with respect to estimated device usage, overall device-related events and event rates for SpaceOAR consistently remained below 1%, with the majority having minimal or no reported clinical impact. The increase in the yearly reported rate is not unexpected given increased use of the device over time. The increase is consistent with previous research which revealed that the number of submissions for all devices to the MAUDE database has grown exponentially since 2001.^27^ In regard to understanding the specific nature of the SpaceOAR MDRs, approximately two-thirds were found to be reported as device malfunction, and approximately one-third were reported as patient injury. Most of the reports were submitted by the manufacturer, highlighting the importance of robust collaboration between the manufacturer and the FDA to promote transparency in reporting for medical devices. The FDA defines device malfunction as a failure of the device in meeting its performance specifications or in performing as intended per the labeling for the device.

Compared to the rates reported above, the observed rate of SpaceOAR-related MDRs is only a small fraction of events that have been assessed prospectively in the literature. To put this in perspective, in a recent retrospective analysis by Yamaguchi et al,^28^ out of 200 consecutive cases, spacers could not be placed in 5% of patients, with the majority of aborted cases because of an unrecognizable fat layer (70%) and challenging anatomy (20%). The authors reported 88% normal placement, 6.3% of patients had rectal wall infiltration, and 1 patient (0.5%) with a suspected prostate subcapsular infiltration. Grade 3 adverse events occurred in 2% of patients; grade 1 events were also uncommon, occurring in 7% of patients. In a subsequent analysis of the postspacer implantation imaging from patients treated on the original pivotal trial by Fischer-Valuck et al.,^29^ rectal wall infiltration was noted in 6% of patients. It was also noted that even with suboptimal spacer gel distribution, significant rectal dose reduction was achieved in 98.7% of cases. In a prospective trial using hydrogel spacers in the setting of stereotactic body radiation therapy to a dose of 45 Gy in 5 fractions, grade 2 acute and late GI toxicities were noted in 23.3 % and 14.3% of patients, respectively, with no grade 3 or higher GI toxicities observed.^30^

In terms of the adverse events resulting in death, a total of 5 events were reported between January 1, 2015 and December 31, 2023, representing 0.002% of units sold over the study period. None of the deaths appear to be related to the SpaceOAR material itself, with most related to cardiovascular effects from procedure positioning and rectal manipulation or from underlying cardiovascular issues. Vasovagal events are a known but uncommon adverse event with transrectal procedures; in a large published experience of over 8000 transrectal biopsy procedures, 0.2% of patients experienced vasovagal syncope.^31^^,^^32^

Two other analyses of SpaceOAR from the MAUDE database have recently been published. Fernandez et al.^21^ reported 574 MDRs identified between June 2015 to October 2022 and Millot et al.^22^ reported 981 MDRs describing 990 events identified between January 2015 to May 2023. Similar to the current study, previous studies showed that the most frequently reported device issue was a device or gel positioning problem. Consistent with our findings, Millot et al. also reported that the second most reported device issue was “adverse event without identified device or use problem,” or an adverse event that occurred with no signs of device malfunction or improper use identified by the reporter. For the most reported patient events, Fernandez et al. reported infection^21^ while Millot et al. reported pain related to the device,^22^ the latter consistent with our findings. Our study identified 5 unique deaths; Fernandez et al. reported 3 deaths while Millot et al. also reported 5 deaths.^1^^,^^21^ Millot et al. also found that malfunction was the most common event type reported and patient injury was the second most common event type, which is in agreement with our findings. There were several methodological differences that may account for differences in results between the studies. While our study and Millot et al. calculated overall rates of MDRs per year using US sales data, Fernandez et al. did not incorporate sales figures. However, Millot et al. calculated yearly rates based on estimated patients treated. A key difference between this study and that by Millot et al is that their study included reports from both US and non-US countries in their MDR and rate calculations, along with not removing duplicates, which may have overestimated MDR events.

The real-world insights gained from this and previous MAUDE analyses complement clinical trial data and other real-world evidence related to SpaceOAR’s device use profile. Clinical trials have shown that hydrogel spacers provide a low morbidity method for reducing rectal toxicity following radiation therapy in men with prostate cancer,^33^ with SpaceOAR demonstrating a well-established effectiveness and safety profile. Clinical trials in the United States^12^, ^13^, ^14^^,^^34^ and Europe^15^^,^^16^ have demonstrated that SpaceOAR hydrogel is safe, easy to use, and that the space created with hydrogel spacers significantly reduces the radiation delivered to the rectum. The randomized SpaceOAR hydrogel US Clinical Trial found that patients who received SpaceOAR hydrogel reported significantly less rectal pain during radiation therapy^2^ and experienced significantly fewer long-term rectal complications.^12^^,^^13^ Current guidelines from the National Comprehensive Cancer Network^35^ and the National Institute for Health and Care Excellence^36^ recommend the use of SpaceOAR hydrogel to reduce rectal toxicity and improve quality of life. Unlike clinical trials, which often have strict inclusion and exclusion criteria, the MAUDE database is a complaint-based surveillance tool, making it challenging to directly contextualize the rates of reported adverse events with broader real-world usage. While the analysis did not include reports outside of the study time frame, ongoing postmarket research, including analyses of large data sets from diverse patient populations, will further our understanding of the optimal application of perirectal spacers.

The strengths of this study are that it is the first study to focus on US reports for SpaceOAR in the MAUDE database, the analyses calculated the overall rate for individual device issues and patient events based on the total units sold in the United States, and duplicate reports were excluded from the data. It must be noted that these US sales figures do not directly equate to the actual number of patients who underwent SpaceOAR placement, as the true case volume is not available. That being said, because of the cost of the SpaceOAR kits, purchasing institutions would be motivated to use them to obtain reimbursement. Similar to other studies using this database, a limitation of this study is the nature of the MAUDE database as a device complaint database that relies on self-reporting, introducing the potential for incomplete or biased data. It is not inherently designed for research purposes; hence, analyses of the MAUDE database do not reflect the overall safety profile of the device and do not constitute risk assessments of the device. Potential database errors and misclassifications within the database could not be discerned and hence could not be rectified. The content of the reports is not extensively validated; there may be more than one report per event, and the severity of the event (apart from the extreme of death or injury) is not rigorously classified. Also, detailed information about potentially important clinical variables and/or pre-existing comorbidities is not available. Therefore, a causal association between the devices and adverse events cannot be proven; it is not possible in all cases to ascertain whether the event occurred because of SpaceOAR, because of radiation therapy alone, or because of the prostate cancer or other patient comorbidities or events. Despite these limitations, the MAUDE database serves as a valuable resource for detecting signals of device-related safety; however, it is crucial to recognize that these reports warrant careful and cautious interpretation and, often, further investigation with additional data sources to fully understand the implications of the reported events.

## Conclusion

This study demonstrated that the reported medical device-related event rate in the US for SpaceOAR remained below 1%, with the majority not being clinically significant. Across 251,836 SpaceOAR and SpaceOAR Vue units sold in the US since 2015, 1005 unique US MDRs were reported, resulting in an overall reported rate across the 9-year study period of 0.40%. When considered alongside the sales volume, this rate is consistent with a relative infrequency of reported issues in the context of extensive SpaceOAR utilization. Although MAUDE is a valuable source of information, there are known limitations, and users should consider all real-world and clinical data to support informed decision-making regarding including this technology in prostate cancer treatment.

## Disclosures

Michael R. Folkert reports article publishing charges and writing assistance were provided by Boston Scientific Corporation. Sirikan Rojanasarot, Emmanuel Ezekekwu, and Danielle Vannan report a relationship with Boston Scientific Corporation that includes: employment and equity or stocks. Young Eun Shin is a graduate student at the University of Cincinnati working on a Global Health Economics and Market Access traineeship with Boston Scientific. Dr Folkert’s institution (University of Washington) is planning to open a prospective trial that is funded by Boston Scientific, Inc. The other authors declare that they have no known competing financial interests or personal relationships that could have appeared to influence the work reported in this paper.

## Appendix Supplementary materials

SpaceOAR MAUDE_ARO_Supplementary Image, application 1

## Footnotes

Supplementary material associated with this article can be found in the online version at doi:10.1016/j.adro.2025.101824.
