# Supplementary material for: Real-World Medical Device Reports of SpaceOAR Hydrogel Spacer: Analysis of the Food and Drug Administration Manufacturer and User Facility Device Experience (MAUDE) Database
Source: Adv Radiat Oncol. 2025 Jun 7;10(12):101824. doi: 10.1016/j.adro.2025.101824 (PMC12661999; doi:10.1016/j.adro.2025.101824)
Supplement: SpaceOAR MAUDE_ARO_Supplementary [file mmc1.docx]

# SUPPLEMENTARY

**Table S1.** **Yearly medical device reports (MDRs), units sold, and reported rates for SpaceOAR using global MDR reports.**

|  | **Total** | | | **SpaceOAR** | | | **SpaceOAR Vue** | | |
| --- | --- | --- | --- | --- | --- | --- | --- | --- | --- |
| **Year** | **MDR** | **Units Sold** | **Rate** | **MDR** | **Units Sold** | **Rate** | **MDR** | **Units Sold** | **Rate** |
| **2015** | 1 | 1,802 | 0.06% | 1 | 1,802 | 0.06% | - | - | - |
| **2016** | 2 | 5,544 | 0.04% | 2 | 5,544 | 0.04% | - | - | - |
| **2017** | 3 | 9,890 | 0.03% | 3 | 9,890 | 0.03% | - | - | - |
| **2018** | 11 | 22,225 | 0.05% | 11 | 22,225 | 0.05% | - | - | - |
| **2019** | 84 | 31,675 | 0.27% | 82 | 31,675 | 0.26% | 2 | - | - |
| **2020** | 126 | 37,155 | 0.34% | 121 | 34,463 | 0.35% | 5 | 2,692 | 0.19% |
| **2021** | 272 | 48,110 | 0.57% | 178 | 30,568 | 0.58% | 94 | 17,542 | 0.54% |
| **2022** | 387 | 50,218 | 0.77% | 129 | 16,840 | 0.77% | 258 | 33,378 | 0.77% |
| **2023** | 323 | 45,217 | 0.71% | 113 | 8,903 | 1.27% | 210 | 36,314 | 0.58% |
| **Total** | 1,209 | 251,836 | 0.48% | 640 | 161,910 | 0.40% | 569 | 89,926 | 0.63% |
